# The Lymphatic System: An Osteopathic Review

**DOI:** 10.7759/cureus.16448

**Published:** 2021-07-17

**Authors:** Raymond J Hruby, Eric S Martinez

**Affiliations:** 1 Neuromusculoskeletal Medicine/Osteopathic Medicine/Family Medicine, Western University of Health Sciences, Pomona, USA; 2 Neuromusculoskeletal Medicine/Osteopathic Medicine, Western University of Health Sciences, Pomona, USA

**Keywords:** osteopathic manipulative treatment (omt), osteopathic manipulative medicine (omm), lymphatics, manual medicine, lymphatic drainage techniques

## Abstract

Osteopathic principles and philosophy suggest the use of osteopathic manipulative treatment (OMT) to restore, augment, or facilitate lymphatic fluid flow to maintain body fluid balance, and/or to stimulate immune system responses to aid in the recovery from illness and maintain normal body defense mechanisms. This review provides an osteopathic view of the role of the lymphatic system in health and disease, with an emphasis on the use of OMT to alleviate somatic dysfunctions (SD) that inhibit the optimum function of the lymphatic system. The current evidence base is reviewed for the use of OMT to assist in restoring or augmenting lymphatic system function to help patients recover from illness and maintain health and wellness. An overview is provided on how osteopathic principles and philosophy relative to the immune system are applied in practice. A literature search was conducted using databases such as Medline, PubMed, Ostmed-DR, and Scopus, focusing on osteopathic approaches to the lymphatic system. Keywords used included osteopathic manipulative medicine, OMT, and lymphatic manual treatment or therapy. Current osteopathic textbook information was also surveyed. There is support for the application of osteopathic principles and OMT for certain conditions that involve the lymphatic system. More prospective research is needed.

## Introduction and background

Osteopathic physicians (DOs) have long recognized how important the lymphatic system is for the maintenance of health. Andrew Taylor Still, the founder of the osteopathic profession, saw the lymphatic system as an absolutely indispensable component of the body and urged physicians not to neglect the system in their treatment of patients. For example, he stated, “We lay much stress on the uses of blood and the powers of the nerves, but have we any evidence that they are of more vital importance than the lymphatics? ... the system of the lymphatics is complete and universal in the whole body” [1]. He further established his view of the importance of the lymphatics when he stated, “We are admonished in all our treatment not to wound the lymphatics, as they are undoubtedly the life-giving centers and organs” [2]. Thus, osteopathic principles and theory place a large emphasis on the lymphatic system’s role in maintaining homeostasis and the functioning interrelationship of all body systems. This article provides an osteopathic view of the role of the lymphatic system in health and disease, with an emphasis on the use of osteopathic manipulative treatment (OMT) to alleviate somatic dysfunctions (SD) that inhibit the optimal functioning of the lymphatic system.

## Review

Definition

The lymphatic system functions as a part of the circulatory system maintaining fluids in the body at balanced levels and as a part of the immune system by playing a role in the body’s defense system against infections. The lymphatic system is comprised of lymphoid organs, lymph tissues, lymph ducts, lymph capillaries, and lymphatic vessels that transport lymph and miscellaneous materials throughout the body [3]. With the discovery of the glymphatic system in the brain, we can now include this network of vessels that serve a similar purpose in the central nervous system [4].

Anatomy of the lymphatic system

The lymphatic system is comprised of lymphatic vessels, lymphoid organs, and lymphatic fluid. DOs utilize certain OMT procedures to stimulate the lymphoid organs, such as the liver and spleen, to enhance the body’s immune system functions to help the patient recover from certain disease states [5-7]. Other OMT procedures are used to either (1) treat SDs that are inhibiting the optimum flow of lymph through the lymph vessels [8,9], or (2) enhance the flow of lymph in order to optimize the delivery of immune system products to the affected body regions during times of illness [10-13].

Lymphatic Vessels

The lymphatic vessels consist of the following structures: lymphatic capillaries, pre-collecting vessels, collecting vessels, lymphatic trunks, and lymphatic ducts.

Lymphatic Capillaries

**Figure 1:**
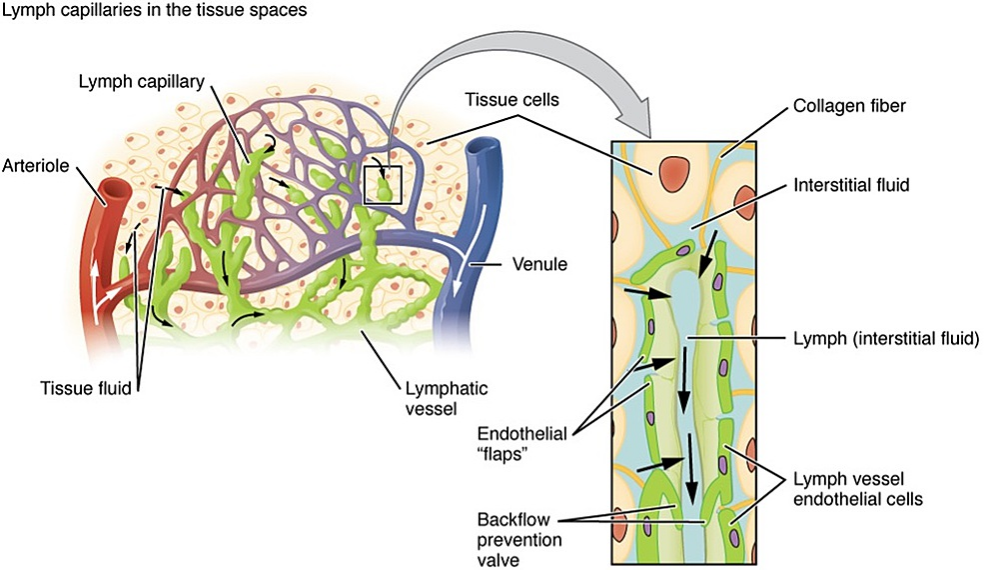
Diagram of a Lymphatic Capillary The lymphatic capillaries run in between the blood vessels of the cardiovascular circulatory system. Adapted from OpenStax College [14].

The lymphatic capillaries’ structure ensures that they can permit the flow of interstitial fluid to absorb proteins and other large molecules (Figure 1) [14]. The walls of the lymphatic capillary, which are made of flat endothelial cells, overlap at their ends in order to allow absorption of interstitial fluid. With increased interstitial pressure, there is a great allowance of fluid entry which is facilitated even further by anchoring filaments which line the capillaries and their surroundings [15]. The lymphatic capillaries are anchored to surrounding tissue by branches of fibroblastic cells which form type VII collagen fibrils [16].

Pre-Collecting Vessels

After the fluid from the interstitium travels through the lymph capillaries or "initial lymphatics," the fluid then travels to the pre-collecting vessels [16]. Like the capillaries of the cardiovascular system, pre-collecting vessels in the lymphatic system have absorption capabilities. These vessels carry lymphatic fluid into the larger surrounding lymphatic vessels [17].

Lymph Collectors

**Figure 2:**
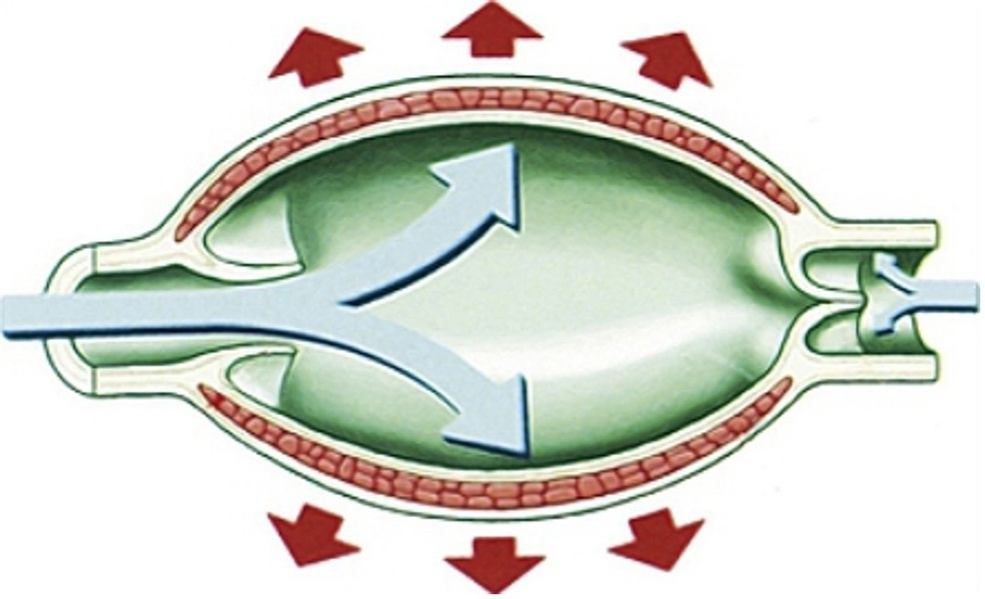
Lymphatic Collector and Lymphangion Adapted from Margaris et al. [18].

In the same way that the pre-collecting vessels are seen as comparable to capillaries, lymph collectors are similar to the veins (Figure 2) [18].

The lymphatic pathways have their own intrinsic active capacity to transport the lymph. Actin filaments have been found in the lymphatic vessels (into smooth muscle cells), which are able to contract and create their own tone. Actin exhibits a transient but constant depolarization due to calcium-dependent chloride currents. This contraction may depend on the sensitivity of actin to feel flow variations (shear stress); the vessels have nerve endings, particularly sympathetic endings, which regulate the contraction of actin filaments [16].

The major differences are that the lymph collectors have less space between their valves and the lymph collectors have slimmer walls than the walls found in veins. The direction of the fluid flow is determined by the passive movement through their one-way valves which prevent backflow of fluid and maintain transport to regional lymph nodes proximally [19]. The lymphatic pathways are able to transport lymph with their own intrinsic active capacity because of actin filaments, which are found in lymphatic vessels, that contract and create their own tone [16].

The lymphangion is the name given to the portion of the collector between a distal and a proximal valve. It is the smallest functional unit of the lymph collector [20]. The lymphangion contains muscle tissue and bicuspid valves, is innervated by the autonomic system, and exhibits intrinsic contractions at a rate of about 6-10 per minute [21]. The intrinsic pacemaker-like contractions are sensitive to hydrodynamic variations, so when flow passes from one lymphangion to another, the pressure gradient is altered and the next functional unit is stimulated and contracts to drain the lymph [16]. 

Lymphatic Trunks and Ducts

**Figure 3:**
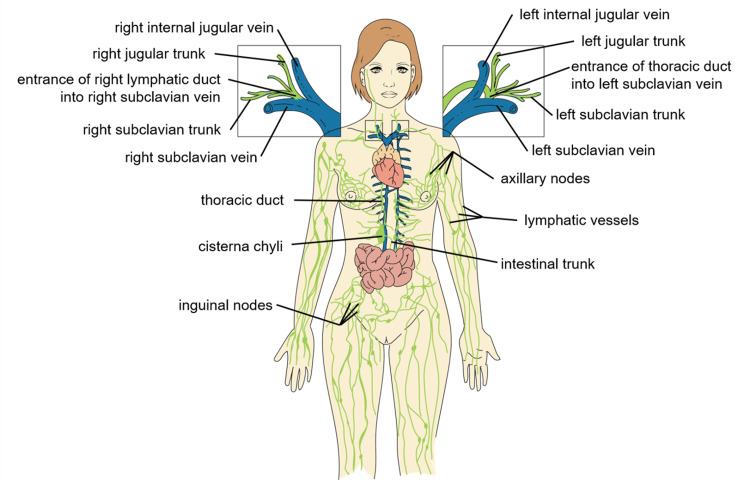
Major Lymphatic Ducts and Trunks Adapted from Menefee et al. [22]. “Lymphatic Vessels, Trunks, and Ducts" by Julie Jenks is a derivative of the original work by Daniel Donnelly and is licensed under CC BY 4.0.

The lymphatic trunks and ducts are the largest of all the lymphatic vessels. While the trunks collect excess fluid from the organs and structures in their localized quadrants, the ducts serve as the vessels that return the lymph back into venous circulation (Figure 3) [19,22].

There are a total of five lymphatic trunks in the lymphatic system. The five lymphatic trunks are as follows: (1) Jugular lymph trunks, which drain lymph from the cervical lymph nodes of the neck; (2) subclavian lymph trunks, which drain lymph from the apical lymph nodes in the armpit; (3) bronchomediastinal lymph trunks, which drain lymph from the regions around the chest including the lungs, heart, and mammary glands; (4) lumbar lymph trunks, which drain lymph from the legs, pelvic region, and kidneys; (5) intestinal lymph trunk which receives chyle from the intestines [23].

**Figure 4:**
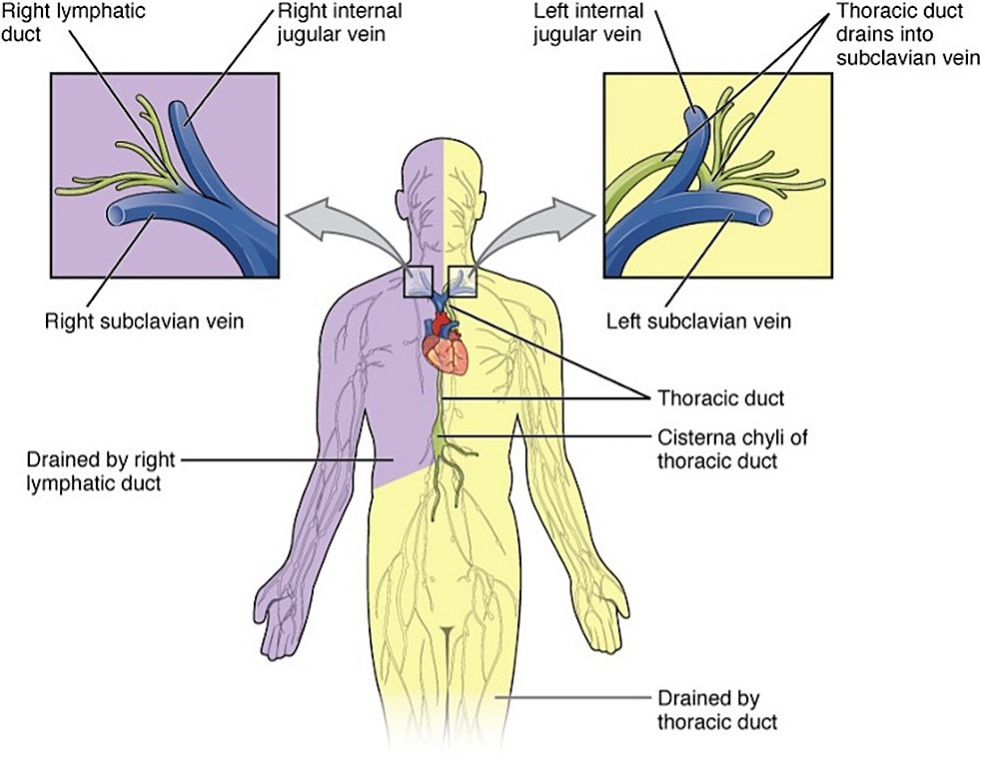
Body Regions Drained by the Thoracic and Right Lymphatic Ducts Adapted from OpenStax College [14].

There are two lymphatic ducts in the body known as the right lymphatic duct and the thoracic duct [24]. The thoracic duct functions as one of the largest channels of the lymphatic system and is responsible for draining up to 75% of the lymphatic fluid into the left jugulovenous angle (left subclavian vein) [25]. Meanwhile, the right lymphatic duct drains a majority of the right upper quadrant into the right jugulovenous angle (right subclavian vein; Figure 4) [14]. 

The glymphatic system

Historically, scientists were not able to identify a lymphatic system in the brain. Intuitively, however, it seemed implausible that the brain would not have a system for the removal of waste products. Finally, in 2012, just such a system was discovered. The name “glymphatic” was given to describe this system. The term is a combination of the words “lymphatic” referring to the function of the system, and “glial,” referring to the type of cells that surround the perivascular spaces through which the cerebrospinal fluid (CSF) flows [26].

**Figure 5:**
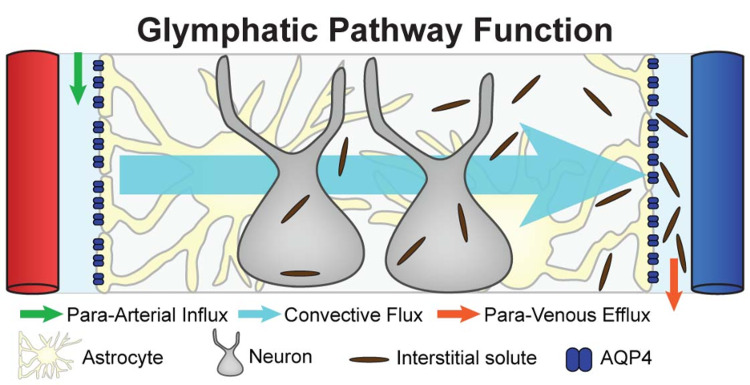
The Glymphatic Pathway Adapted from Iliff [28].

CSF enters the Virchow-Robin space (VRS) which surrounds the walls of paravascular arteries that serve as the blood supply for the brain parenchyma. The exchange of interstitial fluid (ISF) is facilitated by cerebral arterial pulsations and aquaporin-4 (AQP4) water channel proteins [27]. CSF flowing across the VRS allows for interstitial solutes to be deposited and then enter glymphatic vessels in dural sinuses and then drain into lymph nodes, then to lymphatic channels, and finally, the systemic circulation by way of the thoracic duct and the right lymphatic duct (Figure 5) [28].

This knowledge of the glymphatic system is of great interest to osteopathic physicians (DOs). DOs have commonly used OMT to remove impediments, or augment lymph flow, and stimulate the body’s immune system defenses [29]. Knowledge of the role of the glymphatic system will allow DOs to further refine their OMT techniques for the lymphatic system, and their goals of treatment, to further enhance their ability to optimize care for their patients. More effective OMT treatments that address the glymphatic system may prove to be highly useful in the osteopathic approach to conditions like neurogenerative diseases and traumatic brain injury, for example.

Lymphatic fluid

Lymph, also called lymphatic fluid, is normally a clear or yellowish liquid that is slightly less viscous than blood. As the blood circulates, some fluid is lost at the arterial capillary end and becomes interstitial fluid. This interstitial fluid is absorbed into, and removed by, the initial lymphatic capillaries. It continues to pass through a series of increasingly larger collecting vessels, lymph nodes, and ducts and is eventually returned to the blood. Once the interstitial fluid makes its way into the collecting duct, it is referred to as lymph [30].

Lymph consists of water and other substances. These other substances include colloids and minerals, proteins, lipids (absorbed from the intestinal tract and called chyle), cells (such as lymphocytes and macrophages, to name just two), waste products, and other foreign substances. Still, other substances may include (in varying amounts) damaged cells, nutrients, cancer cells, viruses, and bacteria [29,31].

Lymphoid organs

**Figure 6:**
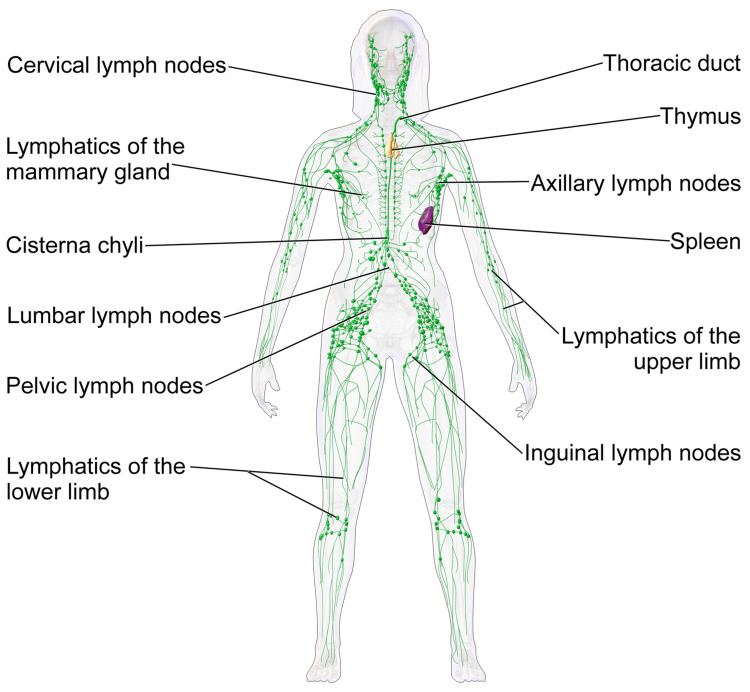
The Lymphoid Organs Adapted from Blausen.com staff [32].

**Figure 7:**
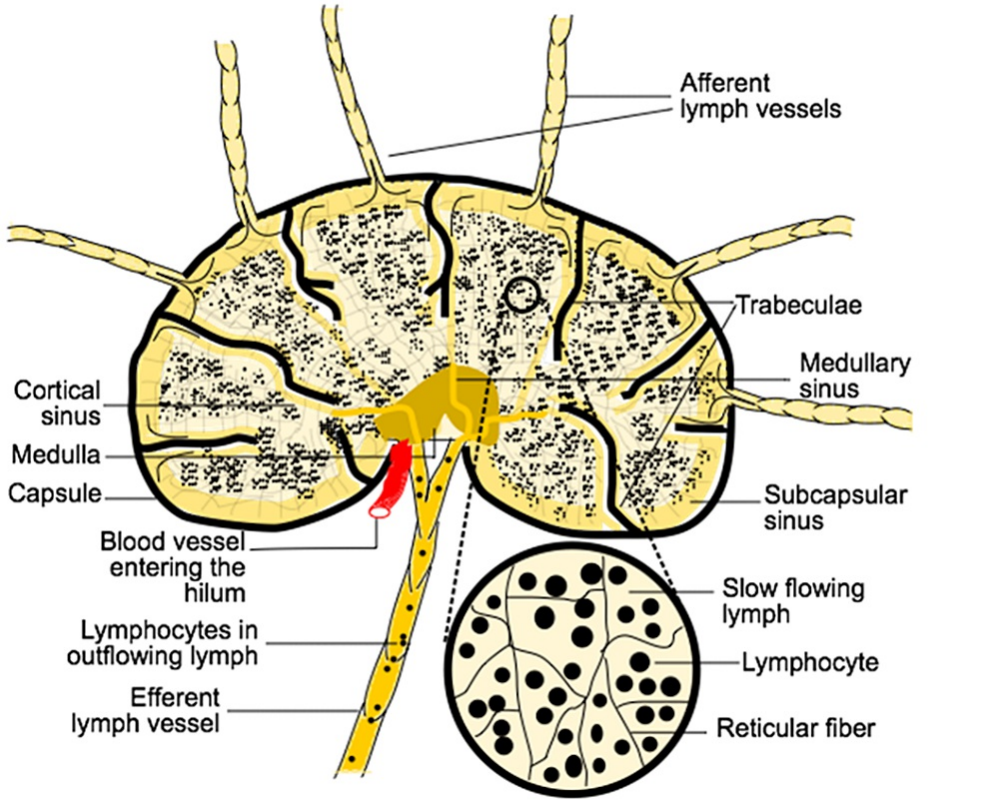
Typical Lymph Node Adapted from Lymph Transport [34].

The following structures are considered lymphoid organs (Figure 6) [32]: the lymph nodes, the spleen, the liver, the thymus, the mucosa-associated lymphoid tissue (MALT), the tonsils, the red bone marrow, and the appendix [33]. The lymph nodes are where the lymph fluid is filtered of toxic and dead material and a place where infection-fighting white blood cells, especially lymphocytes, are stored (Figure 7) [34]. Lymph nodes are also responsible for protein concentration within the lymph [35]. Lymph nodes are found in the head, neck, axilla, and inguinal areas but are mostly found within the intestines at the abdominal level. The spleen helps to filter blood, store blood plasma, accommodates efficient phagocytosis of erythrocytes, and helps induce adaptive immune responses [36]. The thymus is considered both an immunological organ for its role in early life immune system development and as an endocrine gland for its secretion of hormones which play a role in T-cell development and its synthesis of other hormones [37]. The liver produces a large amount of lymph, which is estimated to be 25% to 50% of the lymph flowing through the thoracic duct [38]. The MALT contains a large quantity of the lymphocytes of the immune system and presents as diffuse lymphoid tissue along mucosal surfaces on the body including Peyer’s patches, tonsils, thyroid, breast, lung, salivary glands, eye, skin, and the nasopharynx [39].

Physiologic functions of the lymphatic system

There are three major functions attributed to the lymphatic system. The first major function of the system is the reabsorption and return of excess interstitial fluids into the circulatory system [40]. The lymph capillaries are able to return any excess proteins or interstitial fluid to the venous blood. Second, lacteals, which are lymph capillaries that lie within the villi of the small intestine are capable of absorbing fats and fat-soluble vitamins for metabolism or storage in the bloodstream. Finally, the third major function associated with the lymphatic system is its participation in the body’s immune response and prevention of infectious pathogens. Lymphatic cells known as lymphocytes help to eliminate foreign invading microorganisms. The lymphatic system carries excess interstitial fluid which picks up foreign organisms and other foreign material and it is filtered at the lymph nodes and the lymphatic organs [41].

Physiologic circulation of the lymphatic fluid (lymph flow)

The circulation of the lymphatic fluid begins with the lymphatic capillaries or "initial lymphatics" located within the interstitial fluid space (IFS). The reabsorbed excess fluid, lymph, is then transported to the lymphatic collecting vessels, which contain one-way valves to prevent backflow of lymph. Simultaneously, the lacteals absorb fats and fat-soluble vitamins in the small intestine. Along the way, the lymph passes through one or more groups of regional lymph nodes, which filter the lymph. The next stop will be the lymphatic trunks, and then the left and right thoracic collecting ducts, and finally it enters the left and right subclavian veins, respectively [31].

While in many ways the lymphatic system parallels the cardiovascular system, the lymphatic system does not have its own pump like the heart in the cardiovascular system. Movement in the lymphatic system requires that lymph is moved through the lymphatic system by two types of forces: extrinsic and intrinsic. Extrinsic forces include respiration, the contraction of skeletal muscles that surround the lymphatic vessels, and muscles that act as extrinsic pumps, namely the thoracoabdominal and pelvic diaphragms. Intrinsic forces include spontaneous, pacemaker-like contractions of the smooth muscle walls of larger lymphatic vessels, and stimulation of lymphatic vessel contractility through sympathetic innervation [29,42].

SD and its effects on the lymphatic system

The crucial task attributed to the lymphatic system is the transport of lymphatic fluid from the interstitium to the central circulatory system. The energy for this process comes by way of pumping mechanisms that propel the lymph along with the lymphatic network of vessels. As mentioned above, these pumping mechanisms are extrinsic and intrinsic. The extrinsic pump produces cyclical compression and expansion of lymphatic vessels by such actions as joint movements, muscular contractions, myofascial flexibility, mechanical respiration, and postural changes. The intrinsic pump relies on the rhythmic, spontaneous contractions of lymphatic muscle, and neural modulation via the autonomic nervous system [21].

SD is defined as “impaired or altered function of related components of the somatic (body framework) system: skeletal, arthrodial and myofascial structures, and their related vascular, lymphatic, and neural elements” [29]. SDs that result in restricted joint motion, hypertonic musculature (including restricted thoracoabdominal or pelvic diaphragm motion), abnormal myofascial tension, and/or decreased rib cage motion, may inhibit the cyclical expansion and contraction of the lymphatic vessels, resulting in less than the optimum flow of lymphatic fluid in those regions where SD is present. Any conditions that result in abnormally altered autonomic activity may also reflexively affect the optimum functioning of the intrinsic lymphatic pumping mechanism in segmentally related body regions [29].

Indications

The indications for use of OMT to influence the lymphatic system include, but are not limited to, diseases or conditions that demonstrate some measure of edema, tissue congestion, or lymphatic or venous stasis. Some specific indications are acute SDs, sprains/strains, pregnancy, infection, inflammation, and pathologies with significant venous and/or lymphatic congestion [43,44].

Contraindications

Clinical judgment must be used in employing lymphatic techniques, paying attention to the patient’s diagnosis, clinical condition, and current medical therapy. These factors influence the choice of the appropriate technique, dose, duration, and frequency of treatment.

Absolute contraindications include anuresis if not on dialysis, due to potential risk of pulmonary edema, necrotizing fasciitis (in the area involved), and lack of patient consent and/or cooperation.

Relative contraindications entail the inability of congestive heart failure (CHF) patients to tolerate excessive preload, chronic obstructive pulmonary disease (COPD) due to increased residual volume post-treatment, acute asthma exacerbation due to the risk of increasing reflexive bronchospasm, unstable cardiac conditions, and cancer (immune system activation vs. lymphatic spread). As of today, there are currently no risks associated with lymphatic treatment for patients with cancer, but this area is still under study [29]. Most authorities support judicious use in cancer patients. Treatment over metastatic sites or involved lymph nodes is contraindicated while exercise and OMT to increase lymphatic flow have not been demonstrated to increase cancer risk. The study of lymphatic treatment in the animal model has shown some benefit in pulmonary infectious disease and in pulmonary neoplasm.

Other contraindications include osseous fracture or crushed tissue (in the area involved), bacterial infections with risk of dissemination, chronic infections with risk of reactivation (e.g., abscess, chronic osteomyelitis), diseased organ (i.e., treating thyroid in the presence of hyperthyroidism splenomegaly as in infectious mononucleosis), pregnancy (uterus/deep abdominal work), circulatory disorders (venous obstructions, embolism, hemorrhage), untreated coagulopathies or patients on anticoagulants (modify the technique to be less vigorous), and finally acute herniated nucleus pulposus [43,44].

Safety and efficacy

The safety and efficacy of osteopathic lymphatic treatments have been demonstrated in many published studies without any reports of significant complications [29,44]. It is still essential that the physician practices clinical judgement when prescribing osteopathic lymphatic treatments for their patient.

Diagnostic principles for SD affecting the lymphatic system

To determine the presence of SD and any effects upon the lymphatic system, a systematic approach is helpful. This approach includes the following elements [29,44].

History and Physical Examination

As in any medical or surgical situation, a thorough history and physical examination are essential. The physical examination should give special attention to any evidence of areas of swelling or puffiness, infection, inflammation, organ dysfunction or disease, shortness of breath, and tissue trauma.

Terminal Lymphatic Drainage Sites

**Figure 8:**
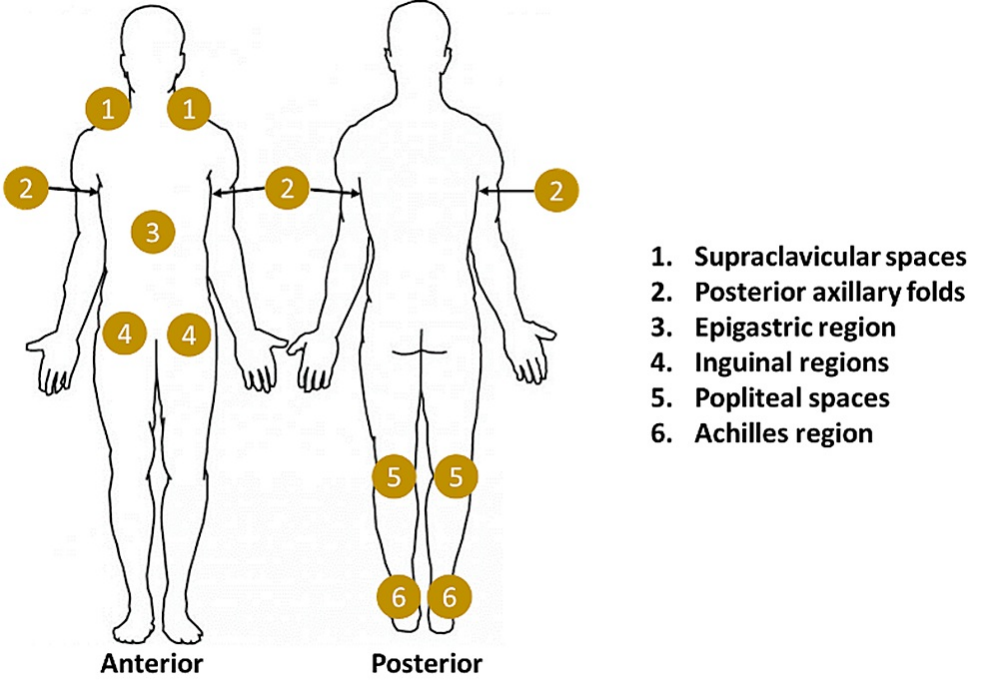
Terminal Lymphatic Drainage Sites

Palpable tension, tenderness or ticklishness, or full, boggy tissue texture changes in the regional terminal lymphatic drainage sites are particularly useful in determining if regional tissue congestion exists (Figure 8).

Central myofascial pathways palpate anatomical transition zones for the presence of fascial restrictions which may inhibit lymphatic flow.

Fluid pumps determine whether the physiologic motion of any of these muscular, fascial, and/or membranous regions is restricted. The cranial dura membranes, especially the tentorium cerebelli (considered by DOs to be a “cranial diaphragm”), the supraclavicular fossa region (often referred to by DOs as the “thoracic inlet”), the thoracoabdominal diaphragm, and the pelvic diaphragm.

Spinal Involvement

SDs of the spinal vertebrae, rib cage, and sacrum result in reduced biomechanical efficiency, and increased tension in their associated muscular and connective tissue structures, all of which may contribute to less optimum lymphatic flow.

Peripheral Pathways

After evaluating terminal lymphatic drainage sites, palpate other regional and local tissues to evaluate the presence of congestion and excess fluid in the interstitial tissues. This helps identify any regions that might benefit from local fluid techniques.

Treatment principles for SD affecting the lymphatic system

Efficient and effective treatment for SD that hinders optimum lymphatic system function must be based on clear treatment goals. Such treatment goals will guide the physician to the most appropriate therapeutic interventions for any given patient, including the use of OMT to help ensure optimum lymphatic system function. Treatment protocols based on clear therapeutic goals should aim to reduce impaired lymphatic fluid movement; enhance the mechanical process of respiration, by reducing the work of breathing and increasing the efficiency of air and fluid flow influenced by this respiratory pump; enhance the movement of lymph and lymphocytes from local, regional, and peripheral tissues that need decongestion [29].

In theory, all OMT techniques can help to optimize lymphatic system functioning through changes in muscle tone, alleviation of myofascial restrictions, modulation of neural reflexes, maintenance of balanced autonomic tone, and beneficial effects on respiration [29].

To establish a rational therapeutic osteopathic approach, DOs use a clinical reasoning process known as the Five Models of Osteopathic Patient Care. The specific models are biomechanical, neurological, respiratory-circulatory metabolic-nutritional, and behavioral-biopsychosocial [45]. Using this framework to guide the information gathering during the history, and physical and structural examinations, the DO can establish a solid foundation for therapeutic decision-making. Some examples of how these models may be viewed with respect to the lymphatic system are explained in Table 1.

**Table 1:** Five Models of Osteopathic Patient Care

Biomechanical model	Restricted joint motions, hypertonic muscles, and increased myofascial tension can inhibit the ability of these external lymph pump components to move lymph fluid and facilitate its return to the central venous system. Diseases or conditions that directly affect the lymphatic vessels may result in a reduction in the optimum function of the intrinsic lymphatic pumps.
Neurological model	Any conditions that disrupt normal autonomic balance and function may also adversely affect the autonomic influence on the lymphatic vessels, again reducing the efficiency of the intrinsic lymphatic pumping mechanisms.
Respiratory-circulatory model	Restricted rib motion and altered intercostal muscle tone, or conditions such as asthma which increases the work of breathing, may affect the ability of this extrinsic pump to assist in the movement of lymphatic fluid. Reduced or inhibited physiologic motion of the thoracoabdominal or pelvic diaphragm, major components of the extrinsic lymphatic pump, will also result in reduced lymphatic fluid flow.
Metabolic-nutritional model	Any diseases or conditions that adversely affect the body’s ability to maintain efficient energy balance may also adversely affect the body’s intrinsic and extrinsic lymph pumps from effectively moving lymphatic fluid. These situations may include those that result in diminished exercise capacity, inability to maintain adequate cardiac output, and nutritional deficiencies. SDs that may contribute to the dysregulation of the body’s ability to produce, distribute and expend energy efficiently may also contribute to diminished lymphatic system functioning.
Behavioral-biopsychosocial model	Optimum care of the patient includes assessing and addressing the patient’s mental, emotional and spiritual needs, along with personal life choices. The DO recognizes that the musculoskeletal system reflects feelings and emotions, and stress can manifest in increased musculoskeletal tension and SD [15]. These excessive stressors may reduce the patient’s resistance to illness or the ability to recover from illness. This may include diminished immune response because of the lymphatic system’s inability to function optimally. Treatment directed at alleviating the patient’s behavioral and psychosocial issues, and the use of OMT to treat the musculoskeletal manifestations of stress (SD) will help to restore the patient to optimum health and wellness.

The use of a treatment protocol

Some DOs prefer to use a more protocol-based approach to the use of OMT to treat SDs that affect the lymphatic system. This method allows for a structured approach to determining which components of the lymphatic system need to be restored to more optimum function, and in what order. In general, treatment addresses the more central components of the lymphatic system and then progresses to the more regional and peripheral components. The principles involved in this approach are [29]: treat the thoracic inlet (supraclavicular fossae) first, to ensure that the areas for terminal lymphatic flow into the central venous system are open; treat SDs of the spinal transitional zones (craniocervical cervicothoracic, thoracolumbar, and lumbosacral junctions) to restore physiologic biomechanical motion in these important spinal regions; treat any restrictions in the diaphragms of the body (cranial, thoracolumbar, and pelvic) which are major pumps for moving lymphatic fluid; treat myofascial restrictions present within affected lymphatic drainage pathways; decongest regional lymph nodes were affected; enhance excessive fluid from any affected peripheral regions.

## Conclusions

From the time of its founding, the osteopathic profession recognized the important role of the lymphatic system. The diagnosis and treatment of the lymphatic system were vital for the maintenance of health and the treatment of disease. This paper has presented a more in-depth exploration of these concepts, demonstrating that the application of osteopathic principles and practices to help restore and enhance immune system functions and lymphatic fluid flow can be an important factor in disease prevention, recovery from illness, and the maintenance of health and wellness.
